# Elastomer with Microchannel Nanofiber Array Inspired by Rabbit Cornea Achieves Rapid Liquid Spreading and Reduction of Frictional Vibration Noise

**DOI:** 10.3390/biomimetics10030164

**Published:** 2025-03-07

**Authors:** Bowen Zhang, Lei Jiang, Ruochen Fang

**Affiliations:** 1 CAS Key Laboratory of Bio-Inspired Materials and Interfacial Science, Technical Institute of Physics and Chemistry, Chinese Academy of Sciences, Beijing 100190, China; 2 School of Future Technology, University of Chinese Academy of Sciences, Beijing 100049, China; 3 International Institute for Interdisciplinary and Frontiers, Beihang University, Beijing 100190, China

**Keywords:** noise reduction, friction-induced self-excited vibration, cornea, microchannels, nanofiber arrays, superspreading

## Abstract

Reducing friction-induced squeal noise is a common issue in daily life and industrial production, particularly for elastomers. However, adjusting structure and wettability in wet environments to solve the friction-induced squeal noise remains a challenge. Here, inspired by rabbit corneas, a microchannel nanofiber array composite structure superhydrophilic elastomer material was prepared to achieve rapid liquid spreading and optimize liquid distribution. Researchers have found that when the depth of the groove microchannel is 400 μm and the length of the nanofiber is 5000 nm, water rapidly spreads on the surface in only 430 ms. This reduces self-excited vibration caused by friction, thereby reducing squealing noise by 20 decibels (dB). This article proposes a novel and direct biomimetic squealing noise reduction strategy, which has great potential in solving friction vibration noise problems in industry and daily life, such as automotive wiper blades, engines, oil lubricated bearings, etc.

## 1. Introduction

Friction frequently occurs in daily life, leading to energy dissipation, with some energy being released as squeal noise [1,2,3]. Self-excited vibration noise resulting from friction is prevalent in daily life, including the operational noise of ships, vehicles, and building equipment. Long-term exposure to squeal noise can significantly impact physical and mental health. In environments requiring high concentration and precise operations, such as aviation, traffic control, and medical operating rooms, squeal noise interference can affect both safety and accuracy [4,5,6]. Over the past few decades, friction-induced squeal noise has been studied by researchers through theoretical analysis, finite element analysis, and other methods. It is currently believed that the generation mechanism of squeal noise primarily includes negative friction-velocity slope, stick-slip, modal coupling mechanisms, and others [7,8,9]. However, few effective squeal noise-reduction materials and strategies have been developed. In previous research, squeal noise reduction strategies such as adding lubricating particles, surface treatment, and internal structure design have primarily been proposed [10,11,12,13,14,15]. Internal structures such as grooves can store friction debris and reduce the generation or propagation of vibration. Under wet friction conditions involving complex interactions such as solid deformation, fluid dissipation, and local flow guidance effects, the friction on patterned surfaces remains inadequately understood, and the associated squeal noise issues have not been thoroughly explored [16]. The microstructure of elastomers significantly influences squeal noise levels. However, the behavior of squeal noise in these elastic microstructures under fluid lubrication remains unexplored. The design and construction of microstructures suitable for friction reduction are challenging, and the development of new squeal noise-reducing elastomers and strategies remains a significant challenge [17]. Natural organisms have evolved unique forms, structures, or surface wettabilities that give them significant lubrication and drag reduction abilities. For instance, shark skin, dolphin skin, penguin feathers, lotus leaves, Nepenthes, and other examples of unique structural lubrication reduce surface resistance [18,19,20,21,22]. Among these, Jiang et al. found that the composite structure of micro groove nanofiber array in rabbit cornea can achieve rapid spreading of liquid, which can make the liquid distribution at the contact interface uniform [23]. These lubrication and drag reduction capabilities offer inspiration for the development of squeal noise reduction strategies in interface friction. Polydimethylsiloxane (PDMS), a silicon-based polymer that is transparent, flexible, and biocompatible, is commonly used for the replication preparation of biomimetic structures [24].

In this study, we mimic the structure of rabbit corneas to prepare microchannel nanofiber composite elastomer materials, modifying the interface structure and wettability to achieve superior liquid spreading performance at the interface. Once the liquid is fully spread at the interface, it mitigates the self-excited vibrations generated by friction, thereby reducing noise. We constructed a physical framework for constant pressure and speed friction noise testing of elastomers in this study. Test results demonstrated that the microchannel nanofiber composite structure, mimicking rabbit corneas, exhibited superior noise reduction performance. By controlling a series of variables, optimal parameters such as spacing and depth were determined for the noise reduction structure, with the highest noise reduction effect reaching.

## 2. Materials and Methods

### 2.1. Materials

The Anodic Aluminum Oxide (AAO) mold was purchased from Shanghai Shang-Mu Nano Technology, Shanghai, China. A SYLGARD silicone elastomer (polydimethylsiloxane) kit with prepolymer and curing agent was purchased from Dow Corning, Midland, MI, USA. Ethanol was purchased from Alfa Aesar, Lancashire, UK. All chemicals for the chemical resistance test were purchased from Sinopharm, Beijing, China. An electricity-driven refrigeration and heating platform, TEC1-26303, was purchased from WAT, P. R. China.

### 2.2. Principle of Biological Super Spreading and Preparation Process of Biomimetic Samples

**Figure 1:**
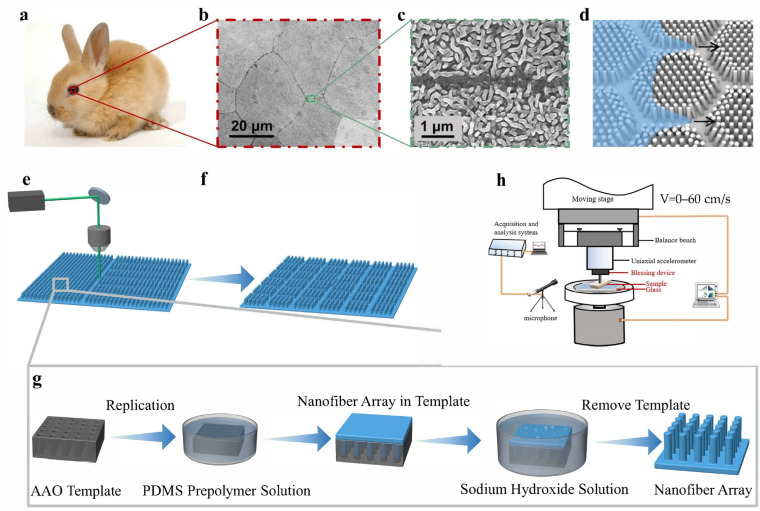
Corneal morphology and preparation of biomimetic rabbit cornea micro-nano composite structures. (**a**–**c**) Optical images and scanning electron microscopy (SEM) images of rabbit corneas are shown. Scanning electron microscopy images reveal that the surface of the rabbit cornea exhibits a uniformly distributed nanofiber array with a diameter of 88.5 ± 3.6 nm, a length of 300–800 nm, and hexagonal microchannels with a side length of 5.5–18.0 µm and a width of 0.47 ± 0.13 µm [23]. (**d**) Schematic diagram of microchannel and nanofiber array morphology, along with the synergistic liquid superdiffusion process on specialized corneas [23]. Reproduced with permission from ref. [23] Copyright © 2021 WILEY-VCH Verlag GmbH. (**e**,**f**) Schematic diagram of the laser etching process for the nanofiber column array structure and the microgroove nanofiber column composite structure. (**g**) Preparation process of the nanofiber array structure. The anodized aluminum oxide (AAO) template is mixed with the PDMS pre-polymerization solution. PDMS has high plasticity and good toughness, making it easy to prepare. It is thermally polymerized to form a nanofiber array elastomer. The AAO template is removed by heating and demolding in a 10% sodium hydroxide solution, followed by cleaning to remove impurities, resulting in a nanofiber array structure PDMS. (**h**) Schematic diagram of friction vibration noise testing device.

The rapid liquid spreading characteristic of the rabbit cornea is attributed to its distinct microchannel and nanofiber array morphology. The corneal surface exhibits multiple polygonal regions (Figure 1a,b), with the intersections of these polygons forming the microchannel structure. The microchannel length ranges from 5.5 to 18.0 µm, with a width of 0.47 ± 0.13 µm. Surrounding the microchannel is the nanofiber array structure, with a diameter of approximately 90 nm and a length ranging from 300 to 800 nm (Figure 1c). Jiang et al. developed a simplified morphological model to explain the rapid liquid spreading characteristics of the rabbit cornea, which posits the existence of periodic polygonal nanofiber arrays and microchannels on the corneal surface (Figure 1d). The nanocapillary forces between the nanofiber arrays facilitate super diffusion, while the microchannels significantly enhance the rate of super diffusion and liquid flow. A 2 µL water droplet can completely spread out to a contact angle of 0° on cornea within 830 ms [23]. To prepare high-toughness microchannel nanofiber array elastomers that mimic rabbit corneas, we employed laser etching of planar PDMS (P-PDMS) and nanofiber array PDMS (NA-PDMS), resulting in microchannel PDMS (M-PDMS) and microchannel nanofiber array composite PDMS structures (MNC-PDMS). By controlling the intensity and duration of laser etching (Figure 1e,f), microchannels of varying depths can be obtained. During the preparation of nanofiber array PDMS, we employed an AAO template and PDMS thermal polymerization replication. First, an AAO template with specific aperture and spacing is selected, placed in a stirred PDMS solution, and evacuated to ensure complete infiltration of the liquid into the AAO template pores. The mixture is then heated in a closed oven for 1 h to polymerize. The prepared nanofiber array PDMS and AAO template are placed together in a 10% sodium hydroxide solution at 60 °C for 12 h, then corroded, demolded, and removed. After removal, residual impurities on the surface of the nanofiber array PDMS are removed using ultrasound to obtain the final nanofiber array PDMS (Figure 1g).

### 2.3. Experimental Setup

To test the frictional vibration noise of P-PDMS, NA-PDMS, M-PDMS, and MNC-PDMS at the wetting interface, we designed a device, shown in Figure 1h, using a Tribometer (Tribometer UMT-2, Bruker Corporation, Wilmington, DE, USA) for constant pressure and speed measurements of frictional vibration noise. In the friction tester, the PDMS sample is placed on a fixed device on the upper sample holder, which is connected to a suspension system and a computer that can accurately control the desired pressure and speed, enabling the two-dimensional moving platform to operate. The disc is installed on the lower sample rack, with glass attached and fixed on top of it. The test sample is pretreated and bonded onto the clamping device. The connecting rod maintains a constant pressure of 20 N on the sample. The test sample undergoes parallel reciprocating motion at speeds ranging from 0 cm/s to 60 cm/s. After starting the Tribometer, the sample adheres tightly to the glass, moves forward a set distance, reverses direction, and returns to its original position, completing one motion cycle. The friction testing process involves over 100 motion cycles, during which water is added to maintain the contact interface in an elastohydrodynamic lubrication environment. A microphone captures audio signals (sensitivity: 50 V/Pa; measurement range: 15–146 dB; frequency response: 3.5 Hz–20 kHz) and transmits them to a computer for analysis of the amplitude and frequency of the emitted sound. The noisy audio is collected using the microphone and analyzed with Adobe Audition and MATLAB R2021a (9.10.0.1602886) (Figure S1). The sampling rate in Adobe Audition is set to 8000 Hz. A period of time (at least four motion cycles) is selected to obtain the noise sound pressure level from 8000 samples per second, and the average noise sound pressure level is then calculated during this period. The average decibel level is then calculated using the following formula, where L represents the decibel level of the sound, P represents the sound pressure level, and P_0_ represents the reference value (usually 20 μPa). The data are represented as the mean ± standard deviation (*n* = 3).

L = 20 × og_10_ (P/P_0_) (1)

### 2.4. Characterization Method

Wetting and superspreading characterizations. Perform contact angle testing using a contact angle meter, place the PDMS structure under test on the testing plane, deposit a 2 μL volume of liquid on the surface, and measure WCA. All values specified in the text are the average of at least five independent measurements. The four materials were treated with oxygen plasma at 200 W for 10 min to induce liquid super-spreading performance on the microstructures. The tests demonstrated that oxygen plasma treatment maintained superhydrophilicity for up to 30 min (Figure S2). Next, 10 μL of ink was applied to observe the liquid spreading behavior on the surfaces of the four materials at 500 frames per second using a high-speed camera.

Structural characterization by SEM. Obtain scanning electron microscopy images using a 10 kV field emission scanning electron microscope (SU8010, Hitachi, Tokyo, Japan). The sample was washed with water, dried with N2, and then a thin layer of gold (EM ACE, Leica, Berlin, Germany) was sputtered to make it conductive for scanning electron microscopy imaging.

Tribological characterization: The tribological experiments were conducted on a Tribometer (Tribometer UMT-2, Bruker Corporation, Wilmington, DE, USA) at 25 °C. In the Tribometer setup, the PDMS sample was fixed on a sample holder attached to the suspension, which allowed movement on a two-dimensional platform. A two-dimensional force sensor (sensitivity: 0.025 N; measurement range: 5–500 N) was installed on the suspension to collect pressure data. A microphone (sensitivity: 50 V/Pa; measurement range: 15–146 dB; frequency response: 3.5 Hz–20 kHz) was fixed on a tripod to measure noise levels. Before the friction test, 10 mL of deionized water was added to the interface.

## 3. Results and Discussion

### 3.1. Surface Morphology and Wettability Characterization

**Figure 2:**
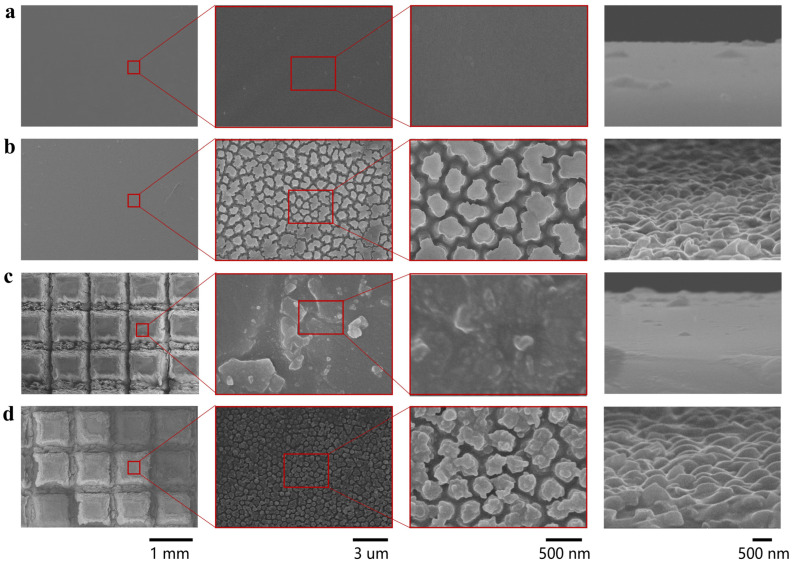
Characterization of the morphology of biomimetic rabbit corneal micro-nano composite structures. (**a**) Scanning electron microscopy (SEM) image of P-PDMS. The PDMS surface with a planar structure exhibits good flatness, with no protrusions observed on the front and side surfaces. (**b**) Scanning electron microscopy (SEM) image of the NA-PDMS. The NA-PDMS exhibits a columnar structure under electron microscopy, with the columns bonded together and showing columnar structures on the sides. (**c**) Scanning electron microscopy (SEM) image of M-PDMS. The M-PDMS and MNC-PDMS formed by laser etching exhibit 1 mm × 1 mm platform under electron microscopy, with the width and depth of the grooves controlled by the laser etching time. (**d**) Scanning electron microscopy (SEM) image of MNC-PDMS. The MNC-PDMS reveals the nanofiber array at the edges, with laser treatment not damaging the nanofiber array structure.

The top and side surfaces of four samples—P-PDMS, NA-PDMS, M-PDMS, and MNC-PDMS—were examined using a scanning electron microscope (SEM). As shown in Figure 2, the surface of P-PDMS exhibits excellent flatness, with no protrusions visible on either the front or side surfaces (Figure 2a). The NA-PDMS displays a columnar structure under electron microscopy (Figure 2b), with nanofiber arrays of varying sizes obtained by using AAO templates of different dimensions (Figure S3). The M-PDMS and MNC-PDMS, formed by laser etching, display 1 mm × 1 mm ridges under electron microscopy (Figure 2c). The ridge height varies with the laser etching time (Figure S4). The groove height after 30 times of laser etching is about 400 μm. The MNC-PDMS reveals the nanofiber array within the platform, and laser treatment does not damage the nanofiber array structure (Figure 2d). SEM characterization confirms the successful preparation of four sample types: P-PDMS, NA-PDMS, M-PDMS, and MNC-PDMS. The laser etching time, spacing, AAO template parameters, and other factors can be precisely controlled. This allows for precise control over the formation of microstructures at the parameter-controlled interface.

**Figure 3:**
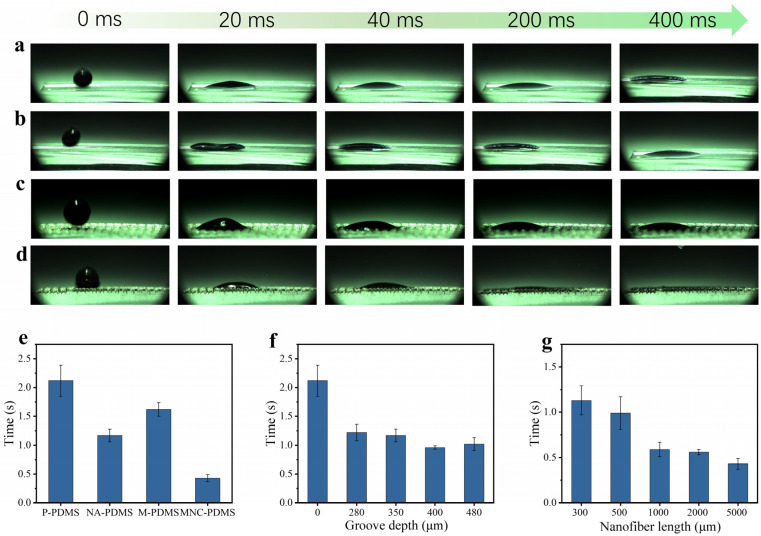
Study on the infiltrative properties of biomimetic rabbit corneal micro-nano composite structures. (**a**–**d**) P-PDMS (**a**), NA-PDMS (**b**), M-PDMS (**c**), and MNC-PDMS (**d**) demonstrate super-spreading properties. The order of spreading speed, from fastest to slowest, is as follows: MNC-PDMS > M-PDMS > NA-PDMS > P-PDMS. The higher diffusion velocity along the microchannel accelerates the diffusion process across the nanofiber array. (**e**) Spreading time of four types of samples: P-PDMS, NA-PDMS, M-PDMS, and MNC-PDMS. Regarding spreading speed, the influence of microchannels is stronger than that of nanofiber arrays, and the combined effect of both structures is more effective. The MNC-PDMS requires only 430 ms to complete spreading. (**f**) The effect of different laser etching depths on spreading speed. The spreading speed of the liquid varies with different etching depths. Test results show that the spreading effect is optimal at a laser etching depth of 400 μm. (**g**) The effect of different heights of nanofiber column arrays on spreading speed. The liquid spreading speed varies with the height of the nanofiber array columns. Test results indicate that a higher nanofiber array column height results in a better spreading effect.

To characterize the liquid spreading performance of the four samples, we conducted wettability tests using a high-speed camera on the following samples: P-PDMS, NA-PDMS, M-PDMS, and MNC-PDMS. It was observed that all four materials exhibited rapid liquid spreading after hydrophilic treatment. Among these, the microchannel nanofiber array composite PDMS achieved liquid spreading within 430 ms, faster than the other three samples. The spreading speed follows the order MNC-PDMS > M-PDMS > NA-PDMS > P-PDMS. The increased diffusion velocity along the microchannel accelerates the diffusion process within the nanofiber array domain (Figure 3a–d). Further research indicates that the height, width, and depth of the microchannels, as well as the nanofiber array, significantly influence the liquid spreading speed. The spreading speeds of the four sample types—P-PDMS, NA-PDMS, M-PDMS, and MNC-PDMS—are compared. In terms of spreading speed, microchannels have a greater influence than nanofiber arrays, and the combined effect of both is superior. The MNC-PDMS completes spreading in only 430 ms (Figure 3e). The spreading speed of the liquid also varies with different etching depths. Test results show that the spreading effect is optimal when the laser etching depth is 400 μm (Figure 3f). The liquid spreading speed is influenced by the height of the nanofiber array columns. Test results indicate that a greater height of the nanofiber array results in a better spreading effect (Figure 3g).

### 3.2. Friction Vibration Noise and Wear Testing

**Figure 4:**
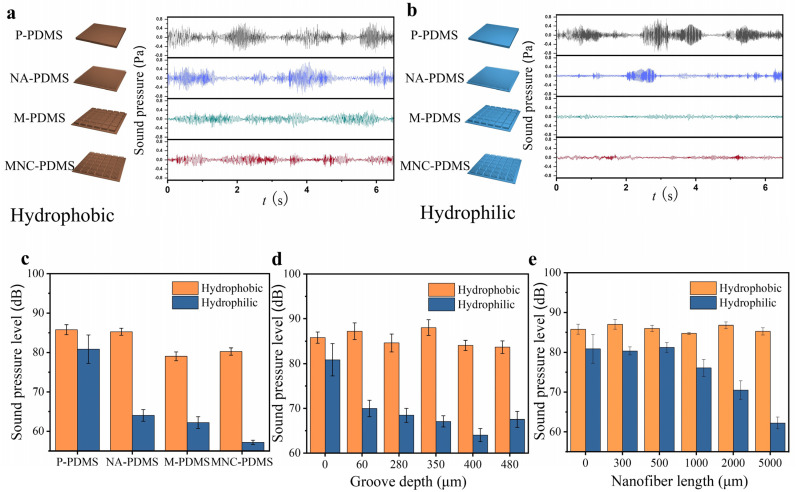
Frictional vibration noise testing of biomimetic rabbit corneal micro-nano composite structures. (**a**) Time distribution waveforms of frictional vibration noise in four hydrophobic states of the following samples: P-PDMS, NA-PDMS, M-PDMS, and MNC-PDMS. The noise distribution is periodic, and the NA-PDMS does not show a reduction compared to P-PDMS. However, the noise reduction observed in M-PDMS and MNC-PDMS is attributed to the microchannel groove structure’s function of trapping friction debris. (**b**) Time distribution waveform of frictional vibration noise after hydrophilic modification of the following samples: P-PDMS, NA-PDMS, M-PDMS, and MNC-PDMS. The noise levels, from highest to lowest, are as follows: P-PDMS, NA-PDMS, M-PDMS, and MNC-PDMS. The sample with the greatest noise reduction effect is MNC-PDMS, corresponding to its exceptional ability to rapidly spread liquid. (**c**) Comparison of average noise pressure levels before and after hydrophilic modification of the following samples: P-PDMS, NA-PDMS, M-PDMS, and MNC-PDMS. (**d**) The effect of different microgroove depths on the average sound pressure level of frictional vibration noise. The noise is minimized when the groove depth is 400 μm. Excessive liquid wetting of the surface leads to decreased lubrication, transitioning into dry friction and amplifying self-excited vibrations. (**e**) The effect of different nanofiber column array heights on the average noise pressure level. Frictional vibration noise varies with the height of the nanofiber array columns. Test results indicate that an increase in nanofiber array column height corresponds to a reduction in frictional vibration noise.

To verify the noise reduction capabilities of samples with superior spreading performance, we conducted frictional vibration noise tests on four samples—P-PDMS, NA-PDMS, M-PDMS, and MNC-PDMS—before and after oxygen plasma treatment. The test results are presented as follows. Before hydrophilic treatment, the noise levels of P-PDMS and NA-PDMS were nearly identical, the noise is about 85 dB, while the noise levels of M-PDMS and MNC-PDMS were approximately 80 dB. This is attributed to the microchannel groove structure’s ability to store friction debris, which impedes rapid liquid spreading on the surface, thereby rendering the nanofiber array ineffective in noise reduction (Figure 4a). Following hydrophilic treatment, the four materials exhibited varying noise levels, ranked from highest to lowest as follows: P-PDMS, NA-PDMS, M-PDMS, and MNC-PDMS. Among these, the hydrophilic MNC-PDMS exhibited stable frictional vibration waves over time, without irregular noise (Figure 4b). Next, we measured the average noise pressure levels of the four samples—P-PDMS, NA-PDMS, M-PDMS, and MNC-PDMS—before and after hydrophilic modification. The sample exhibiting the best noise reduction effect was the MNC-PDMS, which corresponds to its superior liquid rapid spreading ability. This resulted in a reduction of frictional vibration noise from 80 dB to 60 dB (Figure 4c). To investigate the relationship between liquid spreading speed, microgroove depth, and noise, we compared the noise reduction effect of microgrooves at various depths. The results indicated that noise was minimized when the groove depth is 400 μm. We speculate that excessive depth reduces liquid wetting of the surface, leading to dry friction and intensified self-excited vibration, warranting further investigation (Figure 4d). To explore the relationship between liquid spreading speed, nanofiber array size, and noise, we compared frictional vibration noise before and after hydrophilic treatment on samples at five different depths: 300 nm, 500 nm, 1000 nm, 2000 nm, and 5000 nm. We found that as the depth increased, the noise level decreased following hydrophilic treatment, which was attributed to the faster liquid spreading speed (Figure 4e).

**Figure 5:**
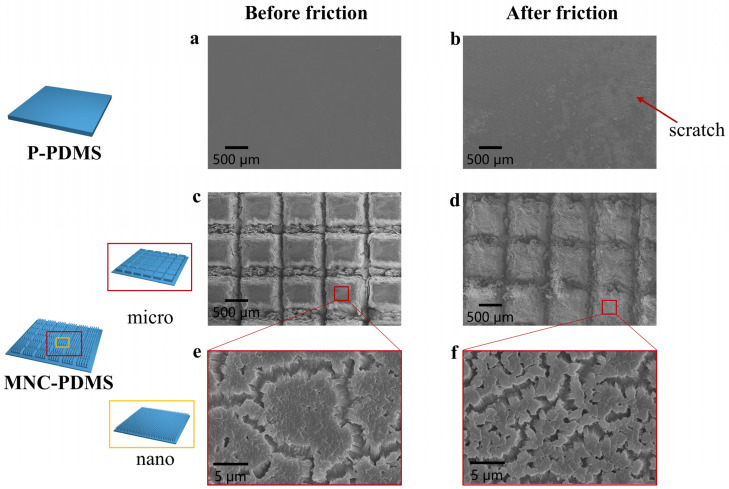
Friction and wear testing of biomimetic rabbit corneal micro-nano composite structures. (**a**,**b**) SEM images of the planar structure before and after the friction test. The surface of the planar structure undergoes self-excited vibration due to intense friction, resulting in both high noise and frictional scratches parallel to the sliding direction. This is caused by the stick-slip phenomenon of the friction contact surface. (**c**,**d**) SEM images of PDMS microgrooves in the composite structure of microchannel nanofiber arrays before and after the friction test. The micrometer-scale structure maintains good morphology before and after friction, with no visible friction marks and reduced self-excited vibration during friction. (**e**,**f**) SEM images of the PDMS nanofiber array composite structure with microchannel nanofiber arrays before and after the friction test. The nanoscale structure retains good morphology before and after friction, without visible friction marks, and with reduced self-excited vibration during friction.

To demonstrate the superior liquid spreading ability of the hydrophilic microchannel nanofiber array composite structure, which can reduce self-excited vibrations caused by friction, we tested the long-term friction and wear performance of the hydrophilic plane and hydrophilic microchannel nanofiber array composite structure. We conducted over 1500 cyclic of friction testing on both the hydrophilic planar structure and hydrophilic microchannel nanofiber array composite structure samples under identical testing conditions. Figure 5 illustrates the surface morphology of the two materials before and after frictional vibration noise testing, as observed using a scanning electron microscope. As shown in Figure 5a,b, after undergoing multiple friction cycles, the planar structure’s surface experiences significant self-excited vibrations due to friction, resulting not only in high noise levels but also in friction-induced scratches aligned with the sliding direction. This phenomenon is attributed to the stick-slip behavior at the friction contact surface, where noise arises from the continuous deformation and rebound vibrations of the material during the unstable sliding process. In contrast, the hydrophilic microchannel nanofiber array composite structure maintains well-preserved micro- and nanoscale morphology before and after friction, with no visible friction marks and reduced self-excited vibrations (Figure 5c–f). In addition, the stretchability properties of P-PDMS, NA-PDMS, M-PDMS, and MNC-PDMS are shown in Figure S5. The Young’s modulus of P-PDMS is 1.50 MPa, and after etching 400 μm, the Young’s modulus of MNC-PDMS is 1.22 MPa; this only decreases by 0.28 MPa, which can be said to not affect the mechanical strength.

## 4. Conclusions

In summary, the unique microchannel nanofiber composite array microstructure on the surface of rabbit corneas facilitates rapid liquid spreading. Inspired by the biomimetic properties of this microchannel nanofiber array, we utilized an anodized aluminum template to replicate and fabricate a noise-reducing elastomer that mimics the micro-nano composite structure of rabbit corneas. Through surface modification, the microchannel nanofiber structure PDMS with rapid liquid spreading ability was prepared. We prepared samples with excellent noise reduction performance by adjusting various parameters. Our results indicate that a microchannel depth of 400 μm, along with specific nanofiber length of 5000 μm, provides the optimal reduction in howling noise, achieving a decrease of 20 dB. This improvement is attributed to the structure’s ability to rapidly guide liquid transport and reduce friction-induced self-excited vibrations. Long term friction and wear tests have shown that PDMS with a biomimetic rabbit corneal microchannel nanofiber array structure can reduce self-excited vibration. The squeal noise reduction strategy proposed in this study effectively addresses the issue of friction-induced noise. The development and application of these novel noise-reducing elastomers can enhance living conditions, protect health, and improve work efficiency. These materials have broad potential for application in reducing noise during strong frictional vibrations in systems such as engines and oil-lubricated bearings.

## Data Availability

Data are contained within the article and Supplementary Materials.

## Supplementary Materials

The following supporting information can be downloaded at: https://www.mdpi.com/article/10.3390/biomimetics10030164/s1, Figure S1: SEM images of top and side view of PDMS with a nanofiber length of 300 nm, 1000 nm and 2000 nm; Figure S2: SEM images of top and side view of Laser etched PDMS morphology and depth. As the laser etching time increases, the etching depth also increases; Figure S3: The changes in the wettability of PDMS treated with oxygen plasma over time; Figure S4: Matlab Code; Figure S5: Stress-strain curves of the P-PDMS, NA-PDMS, M-PDMS, MCN-PDMS; Figure S6: Sound pressure level of Car Wiper and MCN-PDMS; Table S1: Comparison of noise reduction effects of different noise reduction strategies [10,12,15,25,26,27,28].
