# Supplementary material for: Elastomer with Microchannel Nanofiber Array Inspired by Rabbit Cornea Achieves Rapid Liquid Spreading and Reduction of Frictional Vibration Noise
Source: Biomimetics (Basel). 2025 Mar 7;10(3):164. doi: 10.3390/biomimetics10030164 (PMC11940520; doi:10.3390/biomimetics10030164)
Supplement: Supplementary file 1 [file biomimetics-10-00164-s001.zip › biomimetics-3505467-supplementary.pdf]

Elastomer with Microchannel Nanofiber Array Inspired by Rabbit Cornea Achieves Rapid Liquid Spreading and Reduction of Frictional Vibration Noise

Bowen Zhang [1], *Lei Jiang* [1], *and Ruochen Fang* [2, *]

[1]CAS Key Laboratory of Bio-Inspired Materials and Interfacial Science, Technical Institute of Physics and Chemistry, Chinese Academy of Sciences, Beijing 100190, China

[2]International Institute for Interdisciplinary and Frontiers, Beihang University, Beijing 100190, China

Corresponding Authors: Ruochen Fang, E-mail: fangrc@buaa.edu.cn

Supplementary information

```
%% %%%%%%%%%%%%%%%%%%%%%%%%%%%%%%%%%%%%%%% Generate a sine wave signal
[U, fs]=audioread('filename.wav');
x=U(:,1);
x=x-mean(x);
%%%%===========================================
L_T=length(x);
figure;       subplot(211);   plot((1:length(x))/fs,x);
% xlim([1 500+1000]);
xlabel('time[s]');
ylabel('amplitude[Pa]');

%%%%%%%%%%%%%%%%%%%%%%%%%%%%%%%%%%%%%%%% Fast Fourier Transform(FFT)
L_cut=fs;      % The length of the intercepted data is L_cut

novelope=50;

DN=round((1-novelope/100)*L_cut);

N_loop=round((L_T-L_cut)/DN);
%%%%=========================================== Generate frequency axis
df=fs/L_cut;                 % frequency resolution

f=df*(0:L_cut/2);     % Frequency axis corresponding to unilateral spectrum

%%%%===========================================
X_peak=0;
ts=1;
te=L_cut;
%%%%===========================================
for k=1:N_loop

    X_cut=x(ts:te);       % Cut L_cut points for analysis

    L=length(X_cut);
 %%%%===========================================
    U=fft(X_cut);         % FFT

    p2=abs(U/L);

    p1=p2(1:(L/2+1));

    p1(2:end-1)=2*p1(2:end-1);

    X_peak=X_peak *(k-1)/k+p1/k;       % linear average
 %%%%===========================================
    ts=ts+DN;

    te=ts+L_cut-1;
end
%%%%%%%%%%%%%%%%%%%%%%%%%%%%%%%%%%%%%%%%%%%%%%%%%%
max(X_peak)
%%%%%%%%%%%%%%%%%%%%%%%%%%%%%%%%%%%%%%%%%%%%%%%%%%
subplot(212)
plot(f,  X_peak);
xlabel('frequency[Hz]');
ylabel('amplitude[Pa]');

xlim([50 2000])
grid on;
%%%%%%%%%%%%%%%%%%%%%%%%%%%%%%%%%%%%%%%%%%%%%%%%%%
figure;
plot(f,20*log10(X_peak/pref))
xlabel('amplitude[Hz]');
ylabel('sound pressure level[dB]');
xlim([10 1500]);
grid on;
```

Figure S1. Matlab Code.

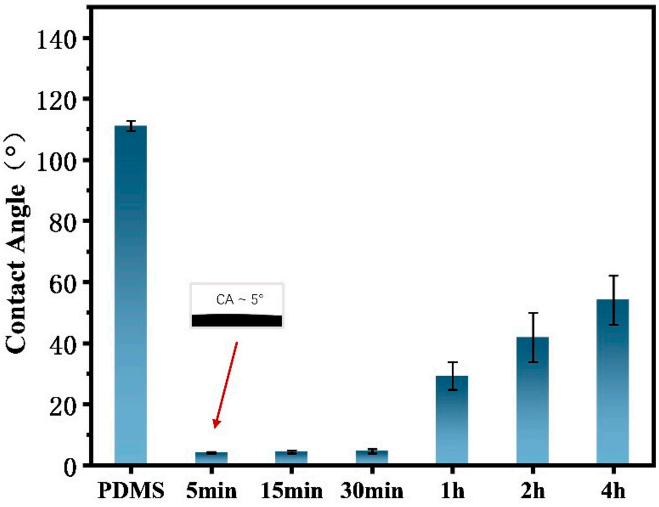

Figure S2. The changes in the wettability of PDMS treated with oxygen plasma over time. Under oxygen plasma treatment, the contact angle changes and the superhydrophilic state can be effectively maintained within 10-30 minutes.

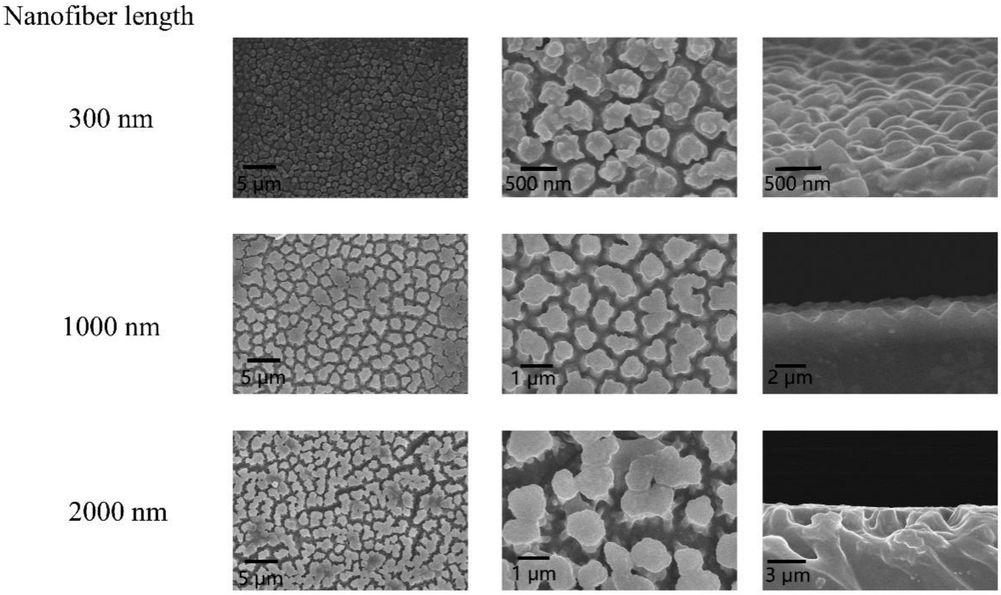

Figure S3. SEM images of top and side view of PDMS with a nanofiber length of 300 nm, 1000 nm and 2000 nm.

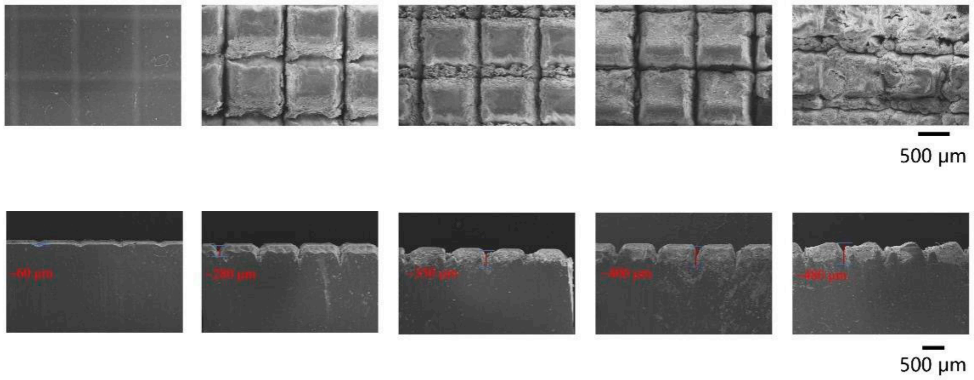

Figure S4. SEM images of top and side view of Laser etched PDMS morphology and depth. As the laser etching time increases, the etching depth also increases.

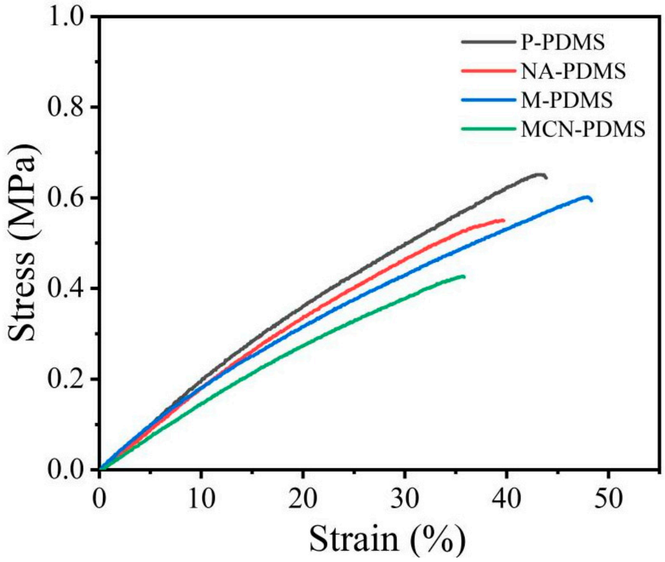

Figure S5. Stress-strain curves of the P-PDMS, NA-PDMS, M-PDMS, MCN-PDMS. The elastic moduli of P-PDMS, NA-PDMS, M-PDMS, and MCN-PDMS are respectively 1.50MPa (P-PDMS), 1.43MPa (NA-PDMS), 1.22MPa (M-PDMS), 1.22MPa (MCN-PDMS).

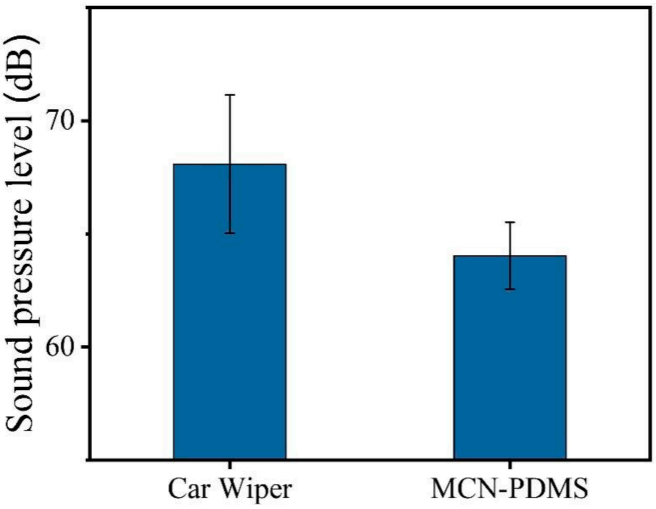

Figure S6. Sound pressure level of Car Wiper and MCN-PD

Table S1 Comparison of noise reduction effects of different noise reduction strategies.

Noise reduction strategy	Specific noise reduction measures	Noise reduction effect	Ref.
Add coating	PTFE Particles	Reduced from 76 dB to 68 dB	[25]
	Bionic Coating	Reduced from 76 dB to 70dB	[26]
Composite material doping	M50 matrix composites by adding Ti_3SiC_2	Reduced from 90 dB to 80 dB	[27]
	Polyimide matrix composite	Reduced from 85 dB to 75 dB	[15]
Surface texture design	Surface roughness	Reduced from 90 dB to 67 dB	[10]
	Groove-textured noise reduction	Reduced from 70 dB to 65 dB	[12]

	Grooves noise reduction	Reduced from 96 dB to78 dB	[28]
This Work	superhydrophilic biomimetics rabbit corneal microchannel nanofiber array PDMS	Reduced from 80 dB to57 dB	-
